# Television viewing and child cognition in a longitudinal birth cohort in Singapore: the role of maternal factors

**DOI:** 10.1186/s12887-019-1651-z

**Published:** 2019-08-16

**Authors:** Ramkumar Aishworiya, Shirong Cai, Helen Y. Chen, Desiree Y. Phua, Birit F. P. Broekman, Lourdes Mary Daniel, Yap Seng Chong, Lynette P. Shek, Fabian Yap, Shiao-Yng Chan, Michael J. Meaney, Evelyn C. Law

**Affiliations:** 1 Department of Paediatrics, Khoo Teck Puat-National University Children’s Medical Institute, National University Health System, 1E Kent Ridge Road, Singapore, 119228 Singapore; 2 Department of Obstetrics and Gynaecology, Yong Loo Lin School of Medicine, National University of Singapore, National University Health System, 1E Kent Ridge Road, Singapore, 119228 Singapore; 3 Singapore Institute for Clinical Sciences, Agency for Science, Technology and Research (A*STAR), 30 Medical Drive, Singapore, 117609 Singapore; 4 Department of Psychological Medicine, KK Women’s and Children’s Hospital, 100 Bukit Timah Rd, Singapore, 229899 Singapore; 5 Duke-NUS Graduate Medical School, 8 College Rd, Singapore, 169857 Singapore; 6 Department of Psychiatry, VU Medical Centre, Amsterdam UMC, VU University, De Boelelaan 1117, 1081 HV Amsterdam, the Netherlands; 7 Department of Child Development, KK Women’s and Children’s Hospital, 100 Bukit Timah Rd, Singapore, 229899 Singapore; 8 Department of Paediatrics, Yong Loo Lin School of Medicine, National University of Singapore, 21 Lower Kent Ridge Road, Singapore, 119077 Singapore; 9 Department of Paediatric Endocrinology, KK Women’s and Children’s Hospital, 100 Bukit Timah Rd, Singapore, 229899 Singapore; 10 Departments of Psychiatry and Neurology & Neurosurgery, McGill University, Montreal, Canada; 11 Sackler Program for Epigenetics and Psychobiology at McGill University, Montreal, Canada; 12 Ludmer Centre for Neuroinformatics and Mental Health, Department of Psychiatry, McGill University, 845 Sherbrooke St W, Montreal, QC, H3A 0G4 Canada

**Keywords:** Television, Screen time, Media exposure, Maternal mental health, Maternal education, Child cognition

## Abstract

**Background:**

Although infant media exposure has received attention for its implications on child development, upstream risk factors contributing to media exposure have rarely been explored. The study aim was to examine the relationship between maternal risk factors, infant television (TV) viewing, and later child cognition.

**Methods:**

We used a prospective population-based birth cohort study, Growing Up in Singapore Towards healthy Outcomes (GUSTO), with 1247 pregnant mothers recruited in their first trimester. We first explored the relationship of infant TV exposure at 12 months and the composite IQ score at 4.5 years, as measured by the Kaufman Brief Intelligence Test, Second Edition (KBIT-2). Multivariable linear regressions were adjusted for maternal education, maternal mental health, child variables, birth parameters, and other relevant confounders. We then examined the associations of maternal risk factors with the amount of daily TV viewing of 12-month-old infants. Path analysis followed, to test a conceptual model designed a priori to test our hypotheses.

**Results:**

The average amount of TV viewing at 12 months was 2.0 h/day (SD 1.9). TV viewing in hours per day was a significant exposure variable for composite IQ (ß = − 1.55; 95% CI: − 2.81 to − 0.28) and verbal IQ (ß = − 1.77; 95% CI: − 3.22 to − 0.32) at 4.5 years. Our path analysis demonstrated that lower maternal education and worse maternal mood (standardized ß = − 0.27 and 0.14, respectively, *p* < 0.01 for both variables) were both risk factors for more media exposure. This path analysis also showed that maternal mood and infant TV strongly mediated the relationship between maternal education and child cognition, with an exceptional model fit (CFI > 0.99, AIC 15249.82, RMSEA < 0.001).

**Conclusion:**

Infant TV exposure has a negative association with later cognition. Lower maternal education and suboptimal maternal mental health are risk factors for greater television viewing. Paediatricians have a role in considering and addressing early risks that may encourage television viewing.

## Background

In the current digital age, children are inevitably exposed to electronic screens regularly and at earlier ages across socioeconomic gradients [1–3]. Numerous studies thus far have shown an association between increased screen time and developmental concerns in young children including language delay, externalising behaviours, and executive functioning deficits [4–7]. A few studies have examined the direct effect of very early screen time on children’s cognition [4, 8–10]. Three earlier studies showed mixed results; one found no significant associations between television (TV) viewing in infancy and visual motor cognition at 3 years of age [8] while two other studies showed delayed cognitive skills in children [4, 9]. The only study that looked at infants 12 months and below showed modest adverse effects of TV on cognitive skills at 14 months [10]. However, this study was completed in a low socioeconomic status population and not in a population-based sample.

Another way to understand the mixed evidence produced by previous studies is to consider potential upstream correlates of high media exposure in young children. One likely risk is family socioeconomic status (SES). Both the Family Investment Theory and the Family Stress Theory support low SES as a risk factor [11]. These theories posit that parents from higher SES households have the ability to provide more learning resources and in-person cognitive stimulation, and may not be as reliant on screens [12] whereas parents from lower SES households as a group face frequent stressors, which may disrupt family routines and shared time with their children, resulting in greater screen time.

A rarely explored risk factor for screen time in infants is maternal mental health. There is clear evidence that children of mothers with depressive and anxiety symptoms have poorer developmental outcomes, e.g., externalizing and internalizing behaviours, academics, compared to children of mothers with little or no mood symptoms [13–16]. Prior research also points to antenatal maternal mood as a stronger correlate of child outcomes than postnatal maternal mood [17, 18]. In fact, our neuroimaging group has shown that antenatal maternal mood alters specific structural brain development of neonates including the amygdala and hippocampus, regardless of postnatal maternal depressive and anxiety symptoms [19, 20]. Little is known, however, about the precise pathways involved in the relationship between maternal mood and developmental outcomes. We hypothesise that media may play a role as an underlying pathway.

Given that the majority of infants are exposed to media use nowadays, [10] exploration of its effect on cognitive skills at this early age is warranted. As infants grow and thrive with responsive interactions and nurturance in their environment for development, it is imperative to study maternal factors in the context of infant media consumption. The aim of this study was to first establish the relationship between TV viewing in infancy and later child cognition in a population-based sample, and secondly, to identify early maternal risk factors for higher screen time. We hypothesize that lower antenatal mood and maternal education are both risks for increased infant TV viewing and for poorer cognitive outcomes. The findings of this study may be leveraged for future interventions, particularly on upstream correlates of negative child outcomes.

## Methods

### Subjects

This is a prospective population-based cohort study with data obtained from the Growing Up in Singapore Towards healthy Outcomes (GUSTO) study [21]. Pregnant women in their first trimester (*N* = 1247) were recruited from two large public hospitals in Singapore, the Kandang Kerbau Women’s and Children’s Hospital and the National University Hospital between June 2009 and September 2010. These mothers belonged to one of the three major ethnicities in Singapore (i.e., Chinese, Malay or Indian). At study baseline, 55.9% of the cohort were Chinese, 26.1% Malay and 18.0% Indian. Study participants were followed after delivery as mother-child dyads. A subset of 423 returned at 4.5 years of age for a battery of neurocognitive tests, including the Kaufman Brief Intelligence Test, Second Edition (KBIT-2), as described below. Assessments on children were completed only in English as the school system in Singapore uses English based bilingual education curriculum. For caregivers whose primary language was not English, back-translated questionnaires were provided. We obtained license agreements with the publishers of copy-righted materials for translation into the local languages. The study was approved by the hospitals’ Institutional Review Board.

### Study measures

Maternal mood was assessed through 3 questionnaires administered to all mothers between 26 and 28 weeks of pregnancy: the Spielberger State-Trait Anxiety Inventory (STAI), the Edinburgh Postnatal Depression Scale (EPDS) and the Beck Depression Inventory, Second Edition (BDI-2). The STAI is a well-studied 40-item measure of state and trait anxiety with a 4 point Likert scale in each question [22].The EPDS is a well-validated measure of depression with 10 items on common depressive symptoms [23]. Similarly, the BDI contains 21 items as a measure of depression [24]. Our group conducted a factor analysis of all the questions in these 3 scales and derived a maternal General Negative Mood Factor [25]. This factor was used as a measure of antenatal maternal mood in this study. Higher values on this General Negative Mood Factor denoted worse maternal mood.

TV viewing was measured through a questionnaire completed by parents in 2010 when the children were 12 months of age. Tablets with touch interface were introduced that year and 97% of families were using TV alone as the main source of screen time for children [26]. Parents reported the amount of TV viewed on weekdays and weekends within the past month. In addition, this questionnaire also asked parents about literacy stimulation activities, for example, reading activities with various caregivers, the presence and frequency of bedtime reading, and the amount of books present at home.

As antenatal maternal mood was included as maternal factors, we used antenatal maternal education as an indicator of SES. Maternal education was categorised into a dichotomous variable with university and above as a group and below university as the other. Variables pertaining to pregnancy and delivery, such as gestational age, birthweight, need for resuscitation, and breastfeeding practices, were systematically collected in this cohort and were adjusted for in the analyses. Children also underwent the Bayley Scales of Infant and Toddler Development, Third Edition, at 24 months of age as part of the cohort measures.

The main outcome measure was the Kaufman Brief Intelligence Test, Second Edition (KBIT-2) administered at 4.5 years, a measure of abbreviated intelligence for children and adults aged 4 years to 90 years of age. The KBIT-2 has been shown to have strong correlations with the Wechsler Intelligence Scale for Children, Fourth Edition (WISC-IV), with a correlation coefficient of 0.84 for the Composite IQ [27]. The verbal components of the KBIT-2 consisted of Verbal Knowledge and Riddles, which measured crystallised (i.e., previously learned) abilities, while the nonverbal component, namely Matrices, measured fluid reasoning. Both the composite IQ and the verbal IQ were examined as separate outcome measures.

### Analyses

We conducted data analysis using IBM SPSS Statistics, Version 22 (SPSS Inc., Chicago, IL) and Mplus (Muthen & Muthen, Los Angeles, CA) [28, 29]. We completed linear regression models using TV viewing in hours per day as the independent variable and child IQ at 4.5 years of age as the dependent variable. We first adjusted for maternal and pregnancy-related variables, then subsequently for child-related variables. A final model was then performed which included all covariates with *p*-value cut-off < 0.1 in the initial models.

**Fig. 1:**
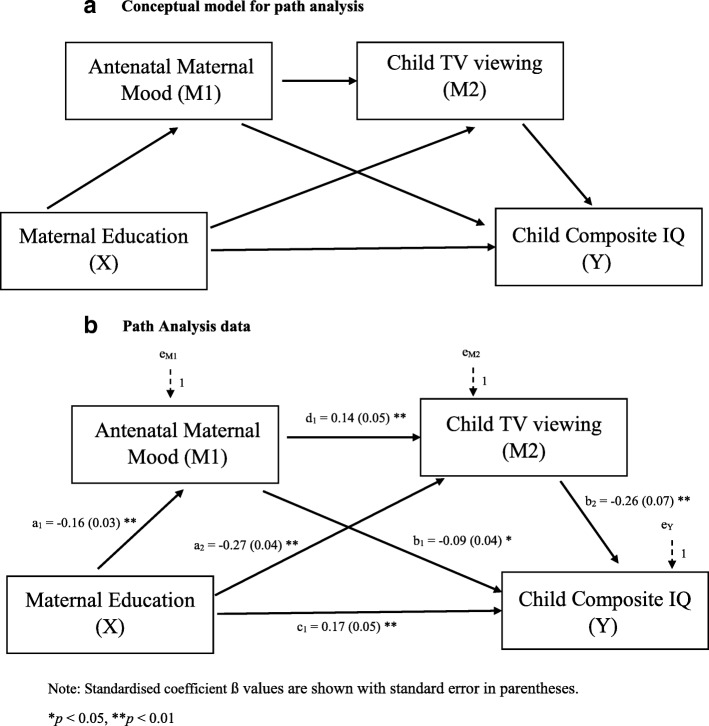
**a** Conceptual model for path analysis. **b** Path Analysis data

The relationships between maternal education, antenatal maternal mood, TV viewing, and cognitive outcomes, were examined using path analysis. Path analysis tested our conceptual model (Fig. 1a) and examined whether TV viewing mediated the effects of maternal variables on child composite IQ. Path analysis was chosen to account for the inter-correlated variables in the model as opposed to simple mediation models*.* The goodness-of-fit of the entire model was evaluated. Four goodness-of-fit indices were examined to determine how well the model reproduced characteristics of the observed data: Comparative fit index (CFI), Akaike’s Information Criterion (AIC), Bayes Information Criterion (BIC), and root mean square error of approximation (RMSEA). CFI and TFI values > 0.95, RMSEA values of ≤0.05, and lower AIC and BIC in the most parsimonious model indicated a good fit [30]. Missing data were addressed using Maximum Likelihood (ML) Estimation in Mplus version 8 [29]

.

## Results

Complete data for all the variables were available for 387 subjects. The average amount of TV viewing at 12 months of age was 2.0 h/day (SD 1.9). Demographic data are as shown in Table 1. We compared children who were part of this current study against the entire GUSTO cohort and found no significant differences in the demographic variables and maternal mood indices on t-tests and chi-square tests. Consistent with previous data from this cohort, [31] there was a significant difference in TV viewing among the 3 ethnic groups with children of Malay ethnicity having more TV viewing compared to those of Chinese or Indian ethnicity (One-way ANOVA F = 9.07, *p* < 0.001).

**Table 1:** Demographic information and descriptive statistics of mothers and children

Variables	Current study cohort		Total GUSTO cohort		*p*-value
	n	%	n	%	
Male gender	212/387	54.8	627 /1176	53.3	0.45
Presence of breastfeeding (1 mth)	370/387	92.2	969 /1050	92.3	0.95
No smoking during pregnancy	325/387	84.0	1052 / 1234	85.3	0.53
Birth Order					
1	184	47.5	541	45.2	0.78
2	114	29.5	414	34.6	
3	59	15.2	172	14.4	
≥ 4	30	7.8	69	5.8	
Maternal Education					
Post-secondary and below	251	64.9	948	67.0	0.10
University and above	136	35.1	466	33.0	
Presence of bedtime reading	123/387	31.8	160 / 538	29.7	0.50
	Mean	SD	Mean	SD	
Prenatal STAI-S score	34.0	9.5	34.8	9.8	0.18
Prenatal EPDS score	7.7	4.3	7.5	4.4	0.35
Prenatal BDI score	8.3	6.2	8.5	6.2	0.60
Maternal General Mood factor	−0.02	0.3	0.00	0.3	0.24
Gestational Age, GA (weeks)	38.8	1.3	38.6	1.6	0.04
Birth weight adjusted for GA z score	−0.03	1.0	0.1	1.2	0.06
Composite score on the Bayley Scales of Infant and Toddler Development at 24 months	102.2	12.6	–	–	
KBIT Composite IQ standard score	92.1	15.0	–	–	
KBIT Verbal IQ standard score	86.0	16.1	–	–	

Notes: *STAI* State Trait Anxiety Inventory, *EPDS* Edinburgh Postnatal Depression Scale, *BDI* Beck Depression Inventory, *KBIT* Kaufman Brief Intelligence Test

Univariate analyses showed that TV viewing in hours/day was a significant predictor of child composite IQ score at 4.5 years of age (ß = -2.72, *p* = < 0.001, 95% CI: − 3.82 to − 1.63). Tables 2 and 3 show the multivariable linear regression results. TV viewing as a linear variable (ß = − 1.55, *p* = 0.02, 95% CI: − 2.81 to − 0.28) and maternal education (ß = 4.78, *p* = 0.04, 95% CI: 0.21 to 9.35) were both significant predictors of composite IQ and verbal IQ at 4.5 years of age. In the final regression model, for every extra hour/day of TV watched, composite IQ decreased by 1.55 standard score points. For example, in a 12-month-old infant who watches 3 more hours/day of TV, the IQ would decrease by 4.5 points in standard scores, which is nearly one-thirds of a standard deviation in the normed sample.

**Table 2:** Linear regression models predicting for composite KBIT score at 4.5 years of age

`	Predictors	ß	*95% CI for B*	*p*-value
Model 1 (Maternal and pregnancy related variables)	Maternal education (Ref: High School and below)	6.51	3.41 to 9.61	< 0.001
	Antenatal maternal mood	−4.81	−9.74 to 0.12	0.06
	Smoking during pregnancy	−4.38	−7.96 to −0.80	0.02
	Birth weight	0.27	−0.85 to 1.40	0.63
	Birth Order	−1.90	−3.26 to −0.53	0.07
	Gestational Age (weeks)	−0.30	−1.31 to 0.71	0.56
Model 2 (child- related variables)	Female gender	1.01	−2.97 to 5.16	0.60
	TV viewing at 12 months (hours per day)	−0.36	−3.69 to −1.29	< 0.001
	Bayley Cognitive score	0.23	0.07 to 0.40	0.006
	Bedtime reading	6.61	2.11 to 11.11	0.004
	Presence of Breastfeeding	3.04	−2.40 to 8.48	0.27
Final adjusted model	**Maternal Education**	4.78	0.21 to 9.35	0.04
	Antenatal maternal mood	−4.76	−12.79 to 3.27	0.25
	Smoking during pregnancy	−4.60	−10.15 to 0.96	0.10
	**Birth order**	−2.27	−4.42 to −0.12	0.04
	**TV viewing at 12 months (hours per day)**	−1.55	−2.81 to − 0.28	0.02
	Bayley cognitive score	0.15	−0.03 to 0.33	0.10
	**Bedtime reading**	6.87	2.25 to 11.48	0.04

Notes: KBIT: Kaufman Brief Intelligence Test; Significant variables are shown in bold

**Table 3:** Linear regression models predicting for verbal KBIT score at 4.5 years of age

`	Predictors	ß	*95% CI for B*	*p*-value
Model 1 (Maternal and pregnancy related variables)	Maternal education (Ref: High School and below)	7.92	4.59 to 11.24	< 0.001
	Antenatal maternal mood	−5.31	−10.60 to −0.02	0.05
	Smoking during pregnancy	−4.67	−8.50 to − 0.82	0.02
	Birth weight	−0.58	−1.79 to 0.62	0.34
	Birth Order	−2.13	−3.60 to −0.67	0.04
	Gestational Age (weeks)	−0.41	−1.49 to 0.68	0.46
Model 2 (child- related variables)	Female gender	2.93	−1.46 to 7.33	0.19
	TV viewing at 12 months (hours per day)	−0.33	−3.60 to −1.00	0.001
	Bayley Cognitive score	0.30	0.12 to 0.48	0.001
	Bedtime reading	6.56	1.70 to 11.43	0.009
	Presence of Breastfeeding	4.81	−1.07 to 10.69	0.10
Final adjusted model	**Maternal Education**	4.94	0.10 to 9.97	0.05
	**Antenatal maternal mood**	−9.83	−18.94 to −0.72	0.04
	Smoking during pregnancy	−0.93	−7.08 to 5.21	0.76
	Birth order	−1.67	−4.05 to 0.70	0.17
	**TV viewing at 12 months (hours per day)**	−1.78	−3.22 to −0.32	0.02
	**Bayley cognitive score**	0.22	0.03 to 0.42	0.03
	**Bedtime reading**	5.36	0.26 to 10.45	0.04
	Presence of Breastfeeding	3.76	−2.49 to 10.01	0.24

Notes: KBIT: Kaufman Brief Intelligence Test; Significant variables are shown in bold

We also completed a separate logistic regression analysis with amount of 12-month TV dichotomised to > 1 h/day and ≤ 1 h/day which showed that the odds of having a composite IQ score < 70 (i.e. less than 2 SD below mean) was 6.2 times higher (95% CI: 1.4 to 27.7) among children who watched > 1 h/day of TV compared to those who watched less than that amount. IQ scores less than 70 meets the IQ threshold for intellectual disability and hence were chosen as the cut-off.

Our path analysis examining the conceptual model (Fig. 1a) demonstrated that lower maternal education and worse maternal mood (standardised direct coefficient − 0.27 and 0.14, respectively, *p* < 0.01) were both risk factors for more TV viewing. There was a serial multiple mediation effect of antenatal maternal mood and amount of TV viewing on the relationship between maternal education and child cognition (Fig. 1b). The indirect pathway through maternal mood alone accounted for 26% of the total effect and the indirect pathways involving TV viewing accounted for 7.9% of the total effect between maternal education and child cognition. The model fit was exceptional with a CFI of > 0.99, AIC of 15,249.82, BIC of 15,310.56 and RMSEA of < 0.001.

## Discussion

Infant television viewing at 12 months of age is negatively associated with cognitive skills at 4.5 years of age. This association remains even after correction for perinatal, child, and family variables. Moving more upstream, our findings demonstrate that lower maternal education and poorer maternal mood are risk factors for greater media exposure in infancy. The pathways through antenatal maternal mood and infant TV viewing strongly mediated one-thirds of the total effect of maternal education on later child cognition.

Consistent with more recent studies on screen time in infants and toddlers, we showed detrimental effects of TV viewing at 12 months on later cognition [4, 9, 10]. Previously published data showed that for every extra hour/day of TV, decreases in working memory, word recognition, and reading comprehension scores were found (i.e., 0.1, 0.3, and 0.6 points, respectively) [9]. Our finding may be specific to our particular culture and population; nonetheless, together with the mounting evidence from other recent studies, it underscores the deleterious effects and the need for guidelines on infant TV adapted to each country. Our findings also reflect poor adherence to the existing guidelines on TV viewing in infants. [32]

The importance of SES on development and cognition has been well established [33, 34]. The underlying mechanisms for this, although not fully elucidated, include home environment and cognitive stimulation [35]. As such, our finding that maternal education is a strong correlate of cognition is not new; however, the finding that TV viewing mediates this relationship is unique. Mediators are good leverage points of intervention and the amount of TV viewing is a modifiable lifestyle change. Although literacy stimulation is not the main subject of this study, we have also shown here that literacy stimulation as measured through bedtime reading is a positive correlate of composite and verbal IQ. This simple, low-cost activity may thus be another potential intervention target to promote cognitive skills.

In line with prior studies, we demonstrate that worse maternal mood is associated with poorer child cognition. This study adds to literature by elucidating one underlying pathway directly and indirectly through TV viewing. Interestingly, poorer antenatal maternal mood has a stronger association with verbal IQ compared to the composite IQ score, which suggests its importance in language and/or crystallized intelligence. It is possible that changes in the brain of these children in utero may affect structures implicated in language pathways. Conversely, antenatal mood may simply reflect postnatal mood. Crystallized literacy knowledge requires caregiver’s interactions with the child, which are likely impaired in mothers with suboptimal mood. Moreover, it is likely that TV viewing acts only as a proxy for reduced direct engagement with the infant. It is important to note that the direct negative effect of maternal mood on child cognition is greater than that through TV viewing, highlighting that media is but one of the pathways in this relationship between maternal mood and child outcomes.

The above results urge medical professionals to actively screen for early family risk factors, namely low family SES and maternal mental health, and to provide anticipatory guidance around infant TV viewing. Addressing these risk factors in a more targeted fashion will ensure that the high-risk groups receive such recommendations.

The limitations of this study include firstly that screen time is limited to TV and does not consider handheld devices and other forms of media. However, at the time of this study, other devices were not in mainstream use. Nonetheless, future studies will encapsulate all other forms of digital media, which has since been collected in the study cohort. Secondly, we did not account for the nature of content viewed on TV. Evidence in older children aged beyond 36 months suggests that educational content and pro-social shows can have positive effects on the child. [36] However, 12-month-old infants have limited ability to process two-dimensional information through the screen regardless of content. Our final cohort size for this study is moderate as opposed to other cohorts examining screen time in children, yet this study is justifiable because maternal and early factors were explored in addition to the impact of screen time on very young children.

## Conclusion

In conclusion, this study confirms the negative relationship between the amount of TV viewing in infancy and cognition in childhood. Lower maternal education and poorer maternal mental health are upstream risk factors for greater TV viewing. This raises important policy implications in terms of identification of specific group of infants who are especially at risk for negative cognitive effects from excessive screen time. It is imperative that paediatricians assess patients for media exposure, especially children from more disadvantaged families and those with mothers facing mental health issues. Parental awareness across the whole population should also be actively encouraged by paediatric and early childhood professionals throughout the community.

## Data Availability

The datasets pertaining to this submitted manuscript are available upon request from the authors.

## Abbreviations

BDI: Beck Depression Inventory
EPDS: Edinburgh Postnatal Depression Scale
GUSTO: Growing Up in Singapore Towards healthy Outcomes
KBIT: Kaufman Brief Intelligence Test
SES: Socioeconomic status
STAI: State-Trait Anxiety Inventory
TV: Television
